# Visual Snow-Like Symptoms and Posterior Uveitis following COVID-19 Infection

**DOI:** 10.1155/2021/6668552

**Published:** 2021-05-29

**Authors:** Kathryn Kelani Braceros, Masumi G. Asahi, Ron P. Gallemore

**Affiliations:** Retina Macula Institute, Torrance, California, USA

## Abstract

Visual snow (VS) is a neurologic condition consisting of a constant positive visual disturbance described as “static” with diagnosis requiring exclusion of competing neurologic and ophthalmologic disorders. The authors describe the first case of visual snow-like symptoms in a patient following coronavirus disease 2019 (COVID-19) infection. He was found to have a transient subtle mild inflammatory reaction in the vitreous and optic nerve edema which resolved, but the VS persisted. Our findings suggest that COVID-19 may precipitate a transient autoimmune response in some patients with resultant ocular inflammation as well as long-term symptoms of VS. This has potential implications for the understanding and treatment of complications related to COVID-19 and in VS.

## 1. Introduction

Visual snow (VS) is a neurologic condition consisting of a constant positive visual disturbance described as “static” or innumerable small dots throughout the visual field [1]. There are often associated visual symptoms such as palinopsia, entoptic phenomena, photophobia, and nyctalopia [1, 2]. The current diagnosis of visual snow requires exclusion of competing neurologic and ophthalmological disorders [2]. Here, we report the first case of visual snow-like symptoms with associated posterior uveitis shortly after coronavirus disease 2019 (COVID-19) infection with persistent VS after resolution of ocular inflammation.

## 2. Case Presentation

The case is a 28-year-old Chinese male with no past medical or psychiatric history presenting with complaints of constant “static” in his entire field of vision which he subsequently described as “a subtle overlay of an out-of-signal TV screen over my entire vision.” Symptoms began shortly following diagnosis with COVID-19 infection. The patient developed symptoms of COVID-19 (fever, cough, loss of sense of taste, and smell) on March 12, 2020, and was confirmed positive for COVID-19 via nasopharyngeal swab on March 20, 2020, by way of a nasopharyngeal swab tested for the coronavirus with reverse transcription-polymerase chain reaction RNA testing. He was subsequently hospitalized for 2 days and treated with IV azithromycin and hydroxychloroquine. The patient was discharged home and eventually tested negative for COVID-19 on April 11, 2020, also via nasopharyngeal swab and RNA, RT PCR. He then reported associated photophobia and palinopsia (after images). He denied prior visual symptoms prior to COVID-19 infection. He was also found to have decreased vision in the left eye by his optometrist and was referred for further evaluation.

**Figure 1:**
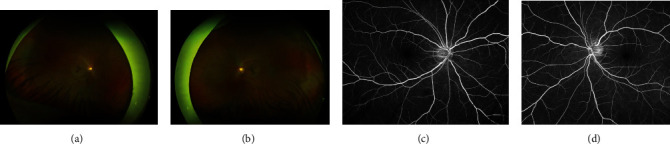
Fundus photo of the (a) right and (b) left eyes at presentation. Late recirculation phase fluorescein angiogram taken at 3 minutes showing subtle staining of the nerve of the (c) right and (d) left eyes.

**Figure 2:**
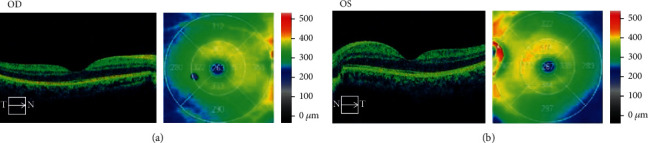
Optical coherence tomography with associated topographical map at presentation showing normal foveal contour with mild macular thickening of the (a) right eye and (b) left eye.

**Figure 3:**
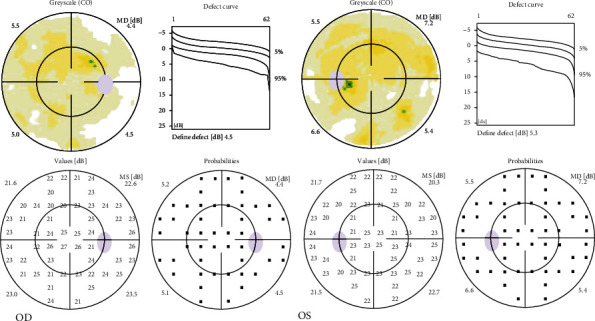
Octopus 30-2 visual field at presentation showing diffuse nonspecific suppression in the right eye (OD) and left eye (OS).

The patient presented with best-corrected visual acuity (BCVA) of 20/20 in both eyes (OU). Intraocular pressure (IOP) was 17 mmHg in the right eye (OD) and 16 mmHg in the left eye (OS). Confrontation visual field was full, extraocular motility intact, and pupils equal reactive and reactive to light. Slit lamp exam (SLE) was with clear lens OU and with rare to trace white blood cells (WBCs) in the vitreous OU, but otherwise unremarkable. The cells were only noted with complete darkness and the slit beam and patient inspection of the vitreous over several minutes. Dilated fundus exam (DFE) was without posterior vitreous detachment (PVD) and retinal tear/break/hole, and the cup to disc (c/d) ratio was 0.6 OD and 0.7 OS (shown in Figures 1(a) and 1(b)). Optical coherence tomography (OCT) of the macula showed mild macular thickening OS (shown in Figures 2(a) and 2(b)). OCT retinal nerve fiber layer (RNFL) analysis showed RNFL thickening OD > OS. Fluorescein angiography (FA) studies showed no overt signs of retinal vascular leakage, but subtle staining of retinal vessels and subtle hyperfluorescence of the disc were present. The Octopus 30-2 visual field showed diffuse suppression OU (shown in Figure 3). Given the subtle inflammatory changes on clinical exam and diagnostic imaging, he was trialed on topical bromfenac 0.075% twice daily (BID) OU.

**Figure 4:**
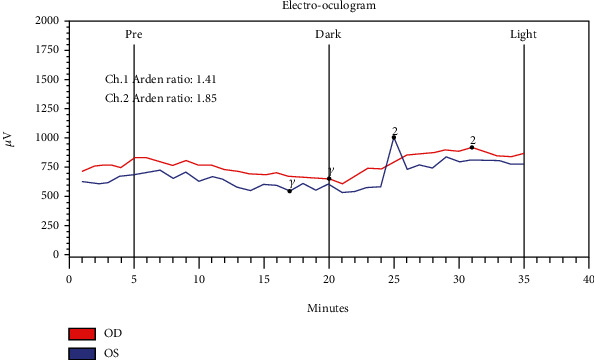
Electrooculogram (EOG) showing suppression in the right eye (OD) and borderline suppression in the left eye (OS) with Arden ratio of 1.41 (OD) and 1.85 (OS).

**Figure 5:**
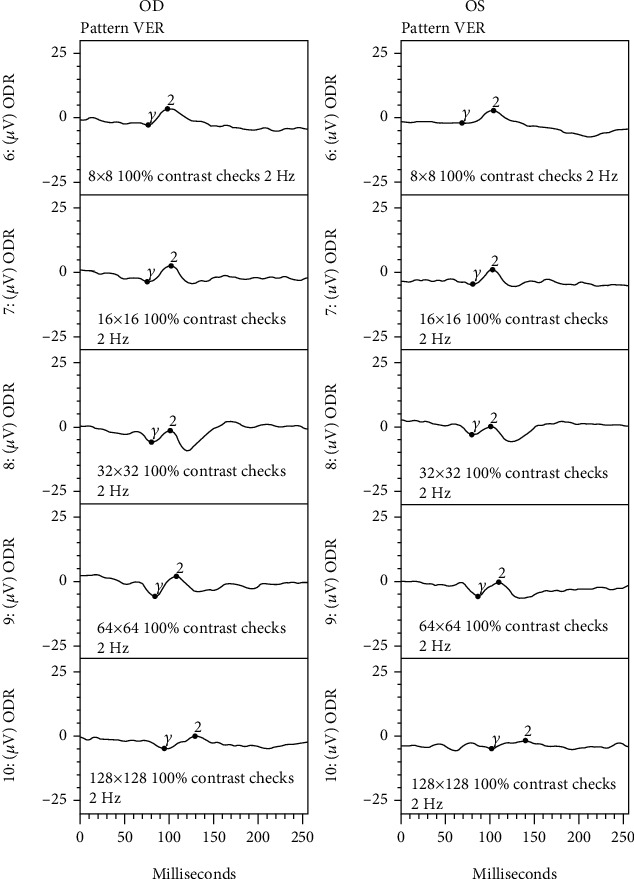
Visual evoked potential/response (VEP/VER) demonstrated mild suppression in the right and left eyes (OD and OS, respectively).

**Figure 6:**
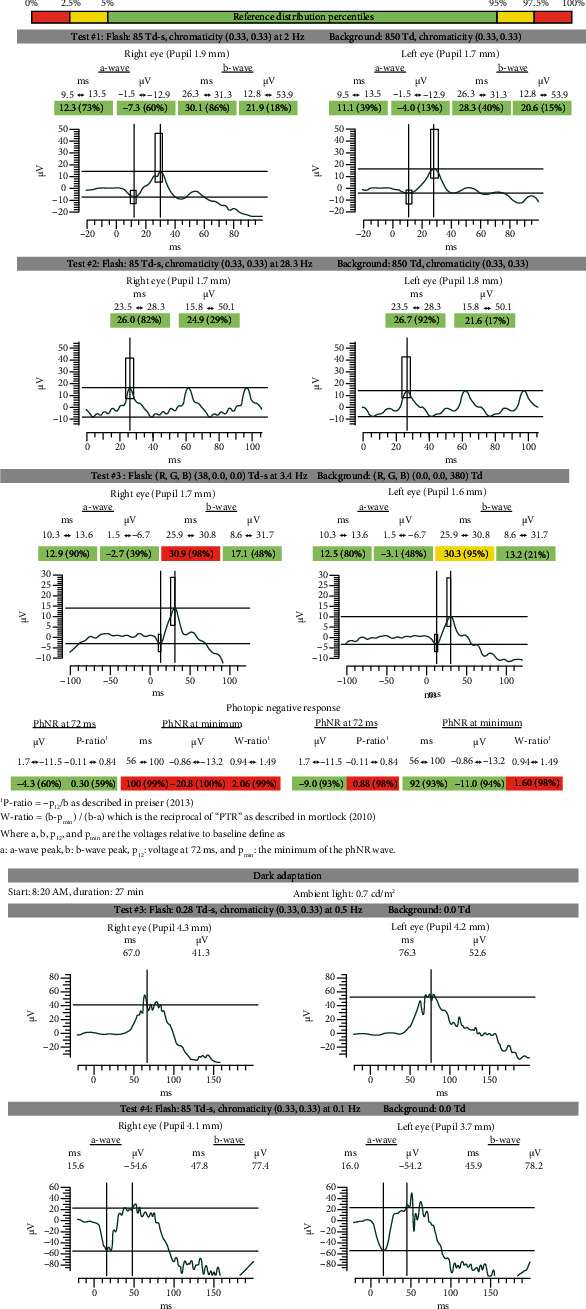
Handheld flash full field electroretinogram (ERG) showing mild to moderate delayed implicit time with reduced photopic negative response OU.

At a subsequent visit, ancillary tests confirmed retinal and optic nerve dysfunction including an electrooculogram (EOG) which showed suppression OD and borderline suppression OS with an Arden ratio of 1.41 (OD) and 1.85 (OS) (shown in Figure 4). Visual evoked potential (VEP) demonstrated mild suppression (shown in Figure 5). Handheld flash electroretinogram (ERG) showed mild to moderate delayed implicit time with reduced photopic negative response OU (shown in Figure 6). The patient underwent laboratory testing with ACE, HLA-B27, ANA, lysozyme, CRP, rheumatoid factor, HIV 1 and 2, ESR, syphilis Ab IgG, QuantiFERON Gold, mitogen, TB1 Ag, TB2 Ag, and CBC, all of which were negative for another cause of ocular inflammation or visual symptoms. Follow-up examination at one month showed decreased retinal thickness consistent with improved posterior segment inflammation but some mild persistent rare cells in the vitreous, and a final follow-up visit three months later revealed resolution of inflammatory cells, stable retinal thickness, and no change in the visual symptoms.

**Figure 7:**
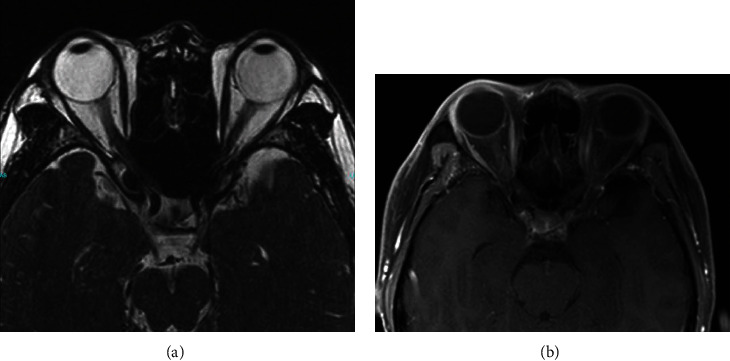
Magnetic resonance imaging (MRI) of head and orbits without evidence of optic nerve abnormality on (a) T2 image and without perineural enhancement of the optic nerve on (b) T1 with contrast and fat suppression.

Magnetic resonance imaging (MRI) of the brain and orbits with and without contrast identified mild sinus disease and an incidental 1.6 cm by 1.7 cm arachnoid cyst in the left anterior temporal region. Orbits were unremarkable, and optic nerve was negative for perineural contrast enhancement (shown in Figure 7). The patient was also seen by a neuroophthalmologist who initially diagnosed the patient with presumed optic neuritis in the left eye despite negative MRI findings but, on follow-up, confirmed the diagnosis of visual snow given the absence of ocular inflammation and other pathology that could account for his symptoms.

The patient did report subjective improvement in symptoms with compliance with topical bromfenac and subjective worsening without the use of the topical bromfenac. Symptoms of “static” persisted on follow-up despite resolution of vitreous cells and a normal ophthalmologic exam. Symptoms continue to date of writing, which is more than ten months since the onset of symptoms.

## 3. Discussion/Conclusion

COVID-19 is an ongoing viral pandemic that is caused by severe acute respiratory syndrome coronavirus 2 (SARS-CoV-2) that is best known for its effect on the respiratory system. Associated symptoms include fever, cough, dyspnea, sputum production, myalgia, arthralgia, headache, diarrhea, rhinorrhea, and sore throat [1]. As clinicians and researchers continue to understand the virus, new clinical manifestations continue to be reported, including neurologic manifestations such as encephalitis, meningitis, acute cerebrovascular disease, and olfactory and gustatory dysfunction [1–5]. Ophthalmic findings have also been reported including anterior uveitis, retinitis, and optic neuritis in animals and hyperreflective lesions at the level of the ganglion cell and inner plexiform layers, subtle cotton wool spots, and microhemorrhages in humans [6, 7].

Emerging reports have also shown COVID-19 preceding the appearance of various autoimmune and autoinflammatory diseases, including pediatric inflammatory syndrome (PIMS) and Guillain-Barre syndrome [8, 9]. This raises questions about the nature of its link with autoimmune and autoinflammatory sequelae. Infectious diseases have long been considered one of the triggers for autoimmune and autoinflammatory diseases, mainly via molecular mimicry, and we hypothesize that an autoimmune response to the recent COVID-19 infection played a primary role in the development of the ocular inflammatory changes noted in our patient [10], specifically the mild vitritis, optic nerve leakage, and mild disc edema as manifested by an increase in the relative nerve fiber layer thickness as well as mild macular edema which manifested as an increase in macular thickness that reduced with treatment with the anti-inflammatory (NSAID) drop, bromfenac.

Posterior uveitis has been reported in patients following COVID-19 infection, and there are reports suggesting retinal inflammation as well. Bakhoum et al. stated that patients have subclinical inflammation in the vitreous detected with SD-OCT, and this is consistent with our findings [11]. There is also a report of intraretinal changes the authors associated with COVID-19 infection, specifically hyperreflective lesions at the level of ganglion cell and inner plexiform layers though subsequent correspondence by others related to the publication questions their origin and significance, raising the possibility of artifact or association with other systemic diseases like diabetes or hypertension [7]. Nonetheless, the report of retinal findings is consistent with our case suggesting posterior segment inflammation associated with prior infection with COVID-19. Of note, animal studies have shown evidence for retinitis and optic neuritis associated with COVID-19, also suggesting retinal involvement with systemic COVID-19 infection [6].

The ocular inflammation in our patient was subtle and difficult to detect—on first glance, the examination appeared normal, but careful inspection of the vitreous under dark-adapted conditions did reveal some cells. Additional data indicated posterior segment dysfunction including the abnormal ERG implicit times for both a- and b-waves, suppressed EOG amplitude, reduced VEP amplitude, generalized suppression of static visual field perimetry, and mild subtle leakage from the optic nerve and retinal vessels on fluorescein angiography. The patient did have a reduction of macular thickness on OCT studies after initiation of treatment with the topical nonsteroidal anti-inflammatory drop, bromfenac, but the visual snow symptoms persisted arguing that the posterior segment inflammation was not the cause of the visual snow symptoms. There is a case of posterior segment inflammation initially diagnosed as visual snow that ultimately was diagnosed as a case of uveitis, birdshot choroidopathy, with resolution of symptoms after treatment [12].

Visual snow has been reported to have a prevalence of 2.2% with symptoms commonly appearing during the late teenage years and early adulthood [13, 14]. The exact etiology has yet to be elucidated. There are strong associations with migraine and tinnitus, suggesting that they may share some common pathophysiologic mechanism with VS [1]. Others have suggested hypermetabolism of the right lingual gyrus, a thalamocortical dysrhythmia, or a feature of hallucinogenic persisting perception disorder [13, 15, 16]. It is a seemingly benign condition, but this condition can be debilitating for patients and may even be the first manifestation of a serious brain disease given a case report confirming Creutzfeldt-Jakob disease on postmortem examination in a patient who developed VS four years after the symptom onset [17]. Given our findings and the others reported above, underlying conditions must be tested before visual snow is made as the diagnosis.

It should be noted that the patient was treated with azithromycin and hydroxychloroquine for COVID-19 prior to presentation to ophthalmology. These medications are not benign and are known to have neurocognitive effects which, however, are less likely contributors to the patient's development of VS. Although clinical trials for azithromycin revealed no neurologic, audiometric, or ophthalmologic side effects [18] [Hopkins], azithromycin has been rarely shown to have cognitive side effects including delirium, disorientation, and impaired concentration typically present within a week of drug ingestion and resolution within three days [19] [Warstler]. Hydroxychloroquine is known to cause retinal toxicity with well-established characteristics and screening guidelines [20] [Marmor]. A recent literature review on hydroxychloroquine found neuropsychiatric effects to be very uncommon with reports of psychosis limited to several case reports [21] [Hamm].

In summary, we present a case of subtle posterior segment inflammation following COVID-19 infections with symptoms suggesting visual snow. Careful examination and ancillary testing confirmed the presence of retinal and optic nerve dysfunction. We propose that an autoimmune response was the culprit. Treatment with anti-inflammatory drops reduced symptoms. This has potential implications for the understanding and treatment of complications related to COVID-19.

## Data Availability

The data used to support the findings of this study are available in the text and figures. Data or figures not included in the manuscript can be made available from the corresponding author upon request.
